# GABA (γ-aminobutyric acid), as a thermo-protectant, to improve the reproductive function of heat-stressed mungbean plants

**DOI:** 10.1038/s41598-019-44163-w

**Published:** 2019-05-24

**Authors:** Manu Priya, Lomeshwar Sharma, Ramanpreet Kaur, H. Bindumadhava, Ramkrishnan M. Nair, K. H. M. Siddique, Harsh Nayyar

**Affiliations:** 1 Department of Botany, Panjab University, Chandigarh, 160014 India; 2 World Vegetable Center, South Asia, ICRISAT Campus, 502 324 Hyderabad, AP India; 3 The UWA Institute of Agriculture, The University of Western Australia, M082, LB 5005, Perth, WA 6001 Australia

**Keywords:** Abiotic, Heat

## Abstract

Rising global temperatures are proving to be detrimental for the agriculture. Hence, strategies are needed to induce thermotolerance in food crops to sustain the food production. GABA (γ-aminobutyric acid), a non-protein amino acid, can partially protect plants from high-temperature stress. This study hypothesises that declining GABA concentrations in the cells of heat-stressed mungbean plants increases the heat-sensitivity of reproductive function. Mungbean plants were grown in a natural, outdoor environment (29.3/16.1 ± 1 °C as mean day/night temperature, 1350–1550 µmol m^−2^ s^−1^ light intensity, 60–65% as mean relative humidity) until the start of the reproductive stage. Subsequently, two temperature treatments were imposed in a controlled environment—control (35/23 °C) and heat stress (45/28 °C)—at about 800 µmol m^−2^ s^−1^ light intensity and 65–70% as mean relative humidity, until pod maturity. In heat-stressed (HS) plants, endogenous GABA concentrations in leaf and anther samples had declined by 49 and 60%, respectively, and to a much lesser degree in the plants, exogenously supplemented with 1 mM GABA. The reproductive function of GABA-treated heat-stressed plants improved significantly in terms of pollen germination, pollen viability, stigma receptivity and ovule viability, compared to untreated HS controls. In addition, GABA-treated heat-stressed plants had less damage to membranes, photosynthetic machinery (chlorophyll concentration, chlorophyll fluorescence, RuBisCO activity were functionally normal) and carbon assimilation (sucrose synthesis and its utilisation) than the untreated HS controls. Leaf water status improved significantly with GABA application, including enhanced accumulation of osmolytes such as proline and trehalose due to increase in the activities of their biosynthetic enzymes. GABA-treated heat-stressed plants produced more pods (28%) and seed weight (27%) plant^−1^ than the untreated controls. This study is the first to report the involvement of GABA in protecting reproductive function in mungbean under heat stress, as a result of improved leaf turgor, carbon fixation and assimilation processes, through the augmentation of several enzymes related to these physiological processes.

## Introduction

Considering the gradual rise in global and local temperatures, heat stress is becoming a major determinant affecting the production potential of various cool-season and summer-season crops^1,2^. Heat stress impairs plant growth and development with marked alterations in phenology, morphology, physiology, biochemistry and gene expression that eventually inhibit the production potential of affected crops^3^. The response of plants to heat stress is dependent on the growth stage; for example, heat stress during the vegetative stage can retard growth, accelerate phenology, and result in chlorophyll loss, scorching and necrosis. Plant cells show damaged membranes, denatured proteins and enzymes in cytosol and organelles, impaired synthesis of carbohydrates and proteins, synthesis of new heat stress-related proteins, oxidative damage, dehydration and loss of turgor-maintaining mechanisms^3^. Heat stress, at the time of reproductive stage, is more detrimental, which can result in flower and pod abortion, and impaired development and function of reproductive components, to severely affect the yield-contributing traits^4^. The male components of reproductive growth (pollen development and function) show more sensitivity than the female components (stigma, style, ovary development and function)^5^. Heat stress can impair fertilisation by obstructing the pollen development, germination and tube growth, resulting in pod set failures^5–7^.

Growth regulating molecules can impart stress tolerance by involving diverse mechanisms^8^. γ-aminobutyric acid (GABA) is a non-protein amino acid that has been implicated as a signalling molecule^9^; the role of GABA in stressed plants has recently received attention^10–12^. GABA levels change in response to stresses and possibly influence the defence mechanisms related to these pathways and processes^9^. GABA has been implicated in plant cell functioning; for instance, it is involved in buffering mechanism in C and N metabolism, regulating cytosolic pH, and protecting from oxidative stress, and is also involved in osmoregulation, and signaling^13,14^. Under heat stress, GABA gets accumulated through calcium-induced activation of enzyme glutamate decarboxylase, as reported in Arabidopsis plants^15^. The role of GABA in sexual reproduction in angiosperms was newly described^16^; along with proline, GABA is indicated as a pivotal amino acid in pollen fertility and vitality^16^. Evidence suggests that GABA is a key determinant of post-pollination fertilisation^16^. The role of GABA in influencing the reproductive function of stressed plants, especially under heat stress, has not been investigated, which formed the basis of the present study.

Mungbean (*Vigna radiata* L.), a summer-season food legume rich in proteins, vitamins and minerals^17^, has a temperature optima of about 35 °C/25 °C (day/night). It often faces heat stress [temperatures >36/28 °C (as day/night temperature)] during the reproductive stage, causing a marked loss of flowers, poor pod set, and reduced pod size, pod and seed numbers and seed yield^6^. Hence, it was considered an ideal plant species to test the thermo-protective effects of molecules, especially GABA. This study aimed to (1) determine the involvement of GABA in mungbean in response to heat stress, (2) probe whether GABA plays a role in protecting the reproductive function of heat-stressed mungbean plants, and (3) find out the mechanisms, possibly associated with protection of reproduction function from heat stress, induced by GABA treatment. It was hypothesised that reduced endogenous GABA levels in Mungbean plants, exposed to heat stress, impair the reproductive function, and that manipulation of these cellular levels, through exogenous means, might reverse the adverse effects of heat stress.

## Results

**Figure 1:**
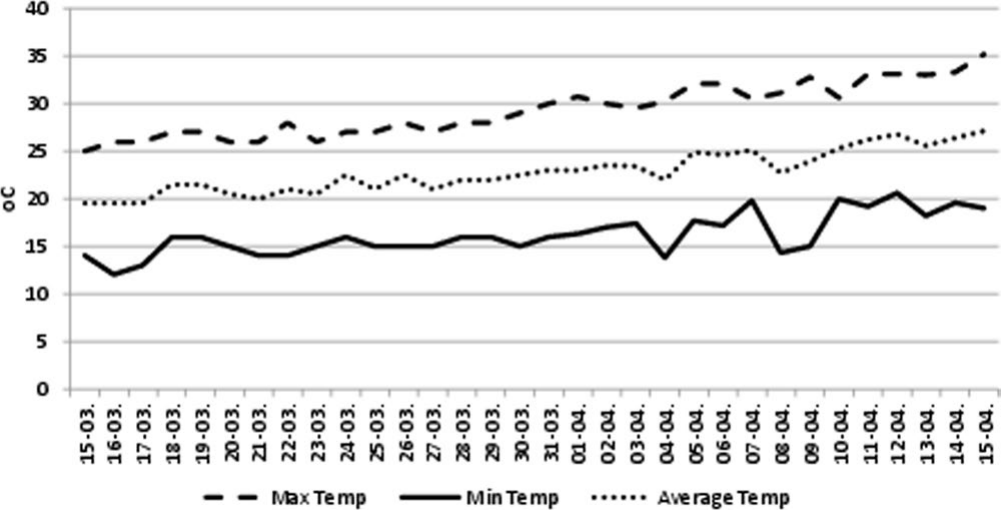
Temperature profile from sowing until the initiation of reproductive stage (March to April).

There were four treatments, as detailed in the Materials and Methods: (a) control (no heat stress or GABA), (b) control + GABA, (c) heat-stress alone, and (d) heat stress + GABA. The leaves and anthers of mungbean plants were tested for various traits, the findings are detailed below. The weather conditions for the plants, which were grown under natural, outdoor environment, before expossing them to heat stress, are given in Fig. 1; the details are given in ‘Materals and methods’ section.

### Endogenous GABA concentration

**Figure 2:**
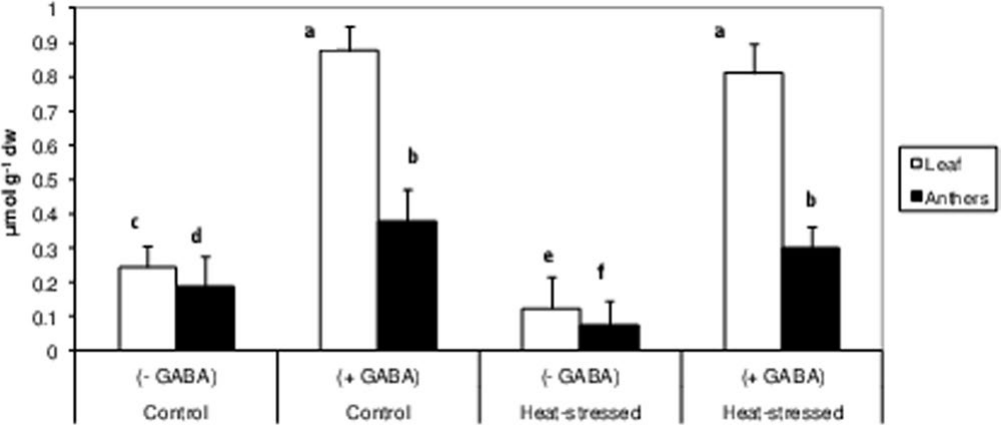
Endogenous GABA concentrations in leaves and anthers of control (without GABA treatment), control (with GABA treatment), heat-stressed (without GABA treatment), and heat-stressed (with GABA treatment) Mungbean plants. Small vertical bars represent standard deviation (n = 3). Different alphabets on bars show significant differences (p < 0.05) from each other.

The leaves and anthers from plants in the four treatments were tested at the flowering stage for endogenous GABA concentrations (Fig. 2). Heat-stress alone reduced endogenous GABA concentrations by 49% and 60% in leaves and anthers, respectively, in comparison to the control plants. Exogenous treatment of GABA substantially increased the endogenous GABA concentrations; by 6.5-fold in leaves and 4-fold in anthers, compared to heat-stressed plants, grown without GABA.

### Membrane integrity

**Figure 3:**
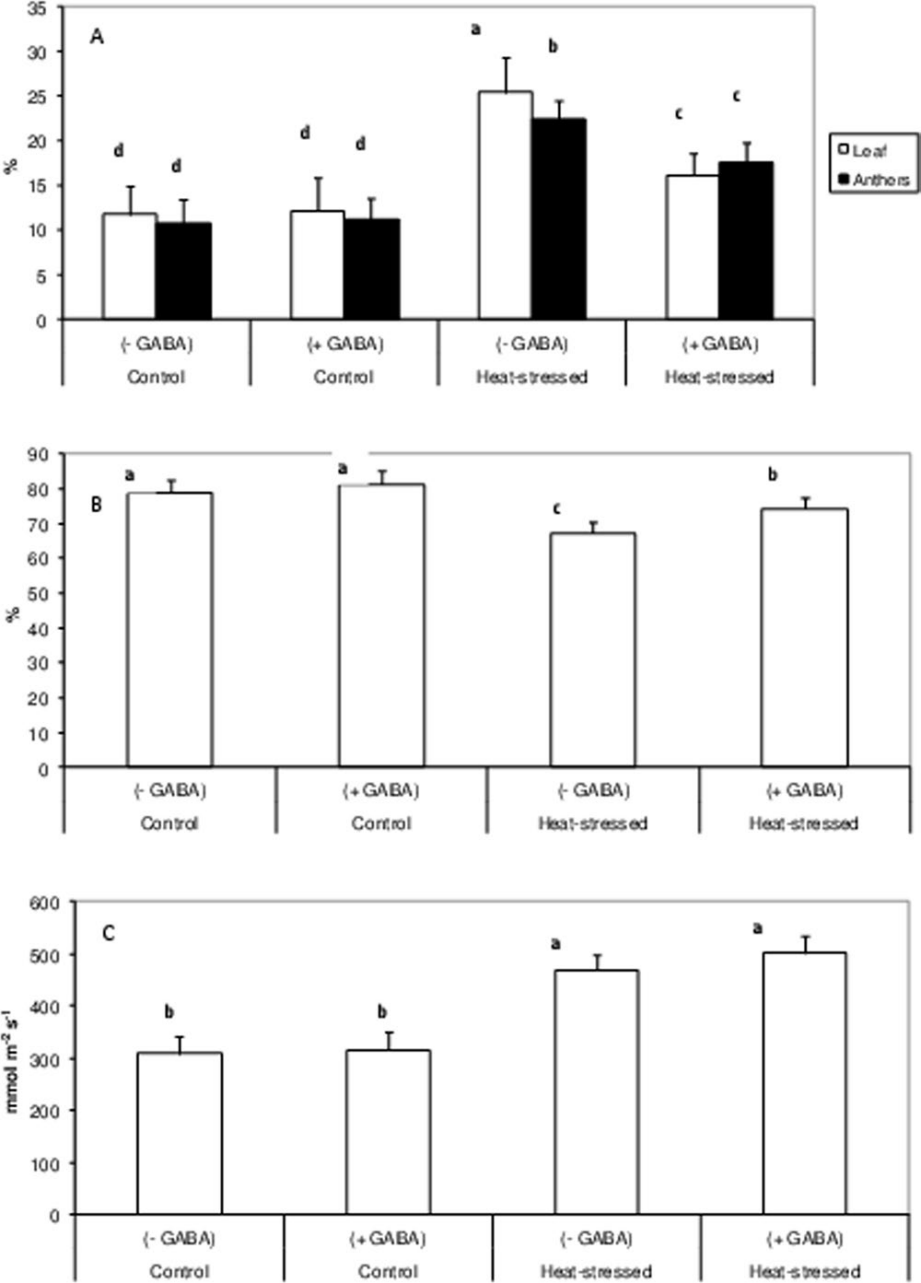
Membrane damage (**A**) in leaves and anthers of control (without GABA treatment), control (with GABA treatment), heat-stressed (without GABA treatment), and heat-stressed (with GABA treatment) Mungbean plants. Relative leaf water content (**B**), stomatal conductance (**C**). Small vertical bars represent standard deviation (n = 3). Different alphabets on bars show significant differences (p < 0.05) from each other.

Heat-stress alone increased membrane damage by more than 2-fold in leaves and anthers, compared to the control (Fig. 3). The control + GABA treatment had little effect on membrane integrity, but the heat stress + GABA treatment reduced membrane damage in leaves (by 1.57-fold) and anthers (by 1.27-fold), relative to heat-stress alone.

### Leaf water status

Leaf water status, measured as RLWC, was 78.9% in the control and 68.7% in the heat-stress alone treatments (Fig. 3). The heat stress + GABA treatment increased RLWC to 75.5%. Stomatal conductance (*g*_*s*_) was 310 mmol m^−2^ s^−1^ in the control and 470 mmol m^−2^ s^−1^ in the heat-stress alone treatments. The heat stress + GABA treatment enhanced *g*_*s*_ to 502 mmol m^−2^ s^−1^ (Fig. 3).

### Reproductive function

**Figure 4:**
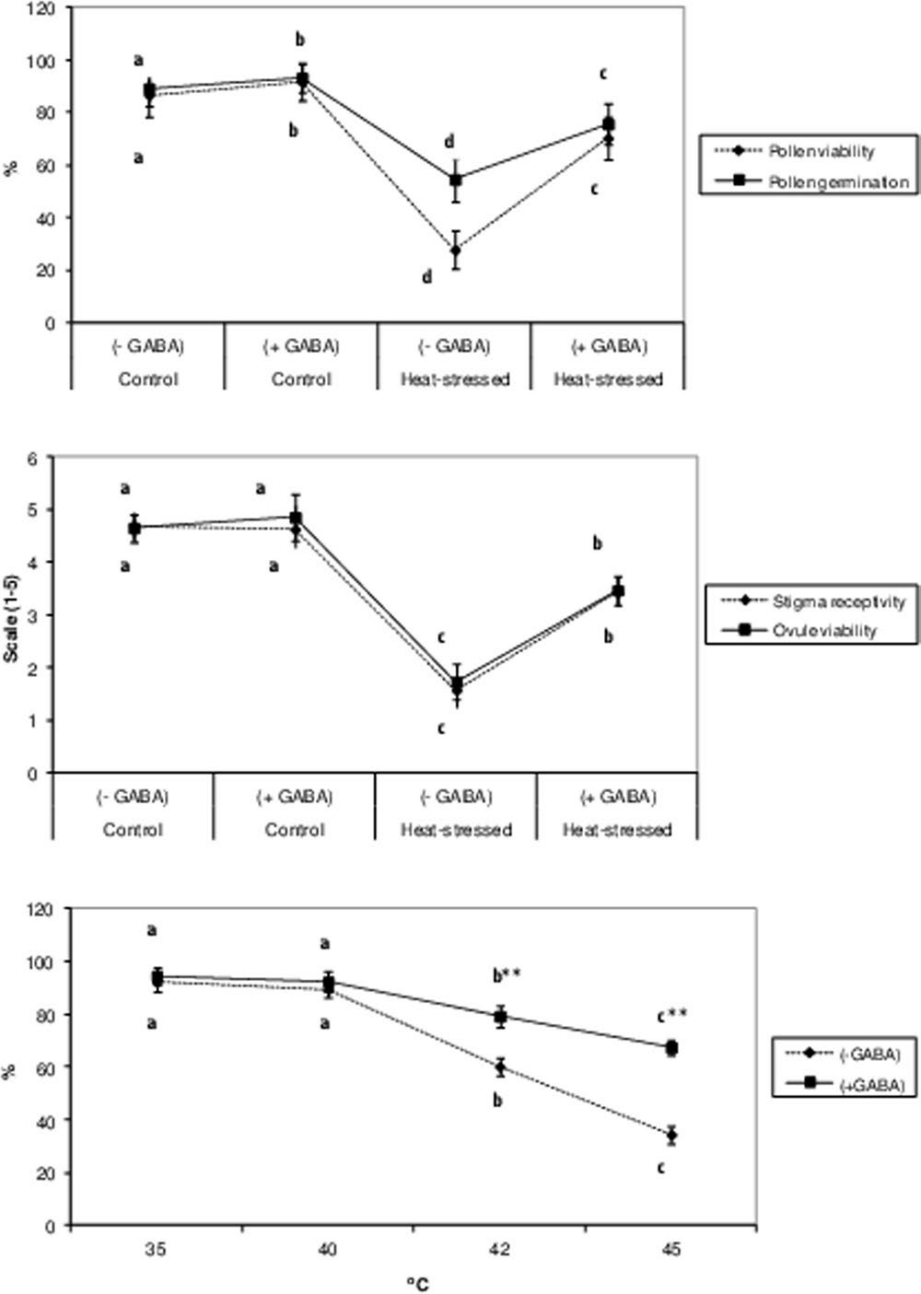
Pollen viability and pollen germination (Top), stigma receptivity, ovule viability (middle) of control (without GABA treatment), control (with GABA treatment), heat-stressed (without GABA treatment), and heat-stressed (with GABA treatment) Mungbean plants. Pollen germination tested with or without GABA at various high temperatures (bottom). Small vertical bars represent standard deviation (n = 3). Different alphabets on bars show significant differences (p < 0.05) from each other.

Pollen viability was 86.65% in the control and 91.65% in the control + GABA treatments (Fig. 4). It declined to 48.4% with heat-stress alone, but only to 70.4% in the heat stress + GABA treatment. Pollen germination (Fig. 4) was 89% in the control plants and 93.3% in the heat-stress alone treatments. The heat stress + GABA treatment increased pollen germination to 75.7%.

Stigma receptivity was 4.69 units in the control and 1.58 units in the heat-stress alone treatments (Fig. 4). The heat stress + GABA treatment increased stigma receptivity considerably, relative to heat-stress alone.

Ovule viability was 4.6 units in the control plants and 1.73 units in the heat-stress alone treatments (Fig. 4). The heat stress + GABA treatment increased the ovule viability to 3.47 units.

Pod set (%) was 74% in the control and 85.3% in the control + GABA treatments. Heat-stress alone reduced pod set to 36.2% while the heat stress + GABA treatment improved pod set to 59.5%.

In a laboratory experiment, pollen germination declined from 92% at 35 °C to 60% at 42 °C and 34.5% at 45 °C (Fig. 4). In GABA-supplemented media, pollen germination increased to 79.3% at 42 °C and 67.5% at 45 °C.

### Photosynthetic ability, carbon fixation and assimilation

**Figure 5:**
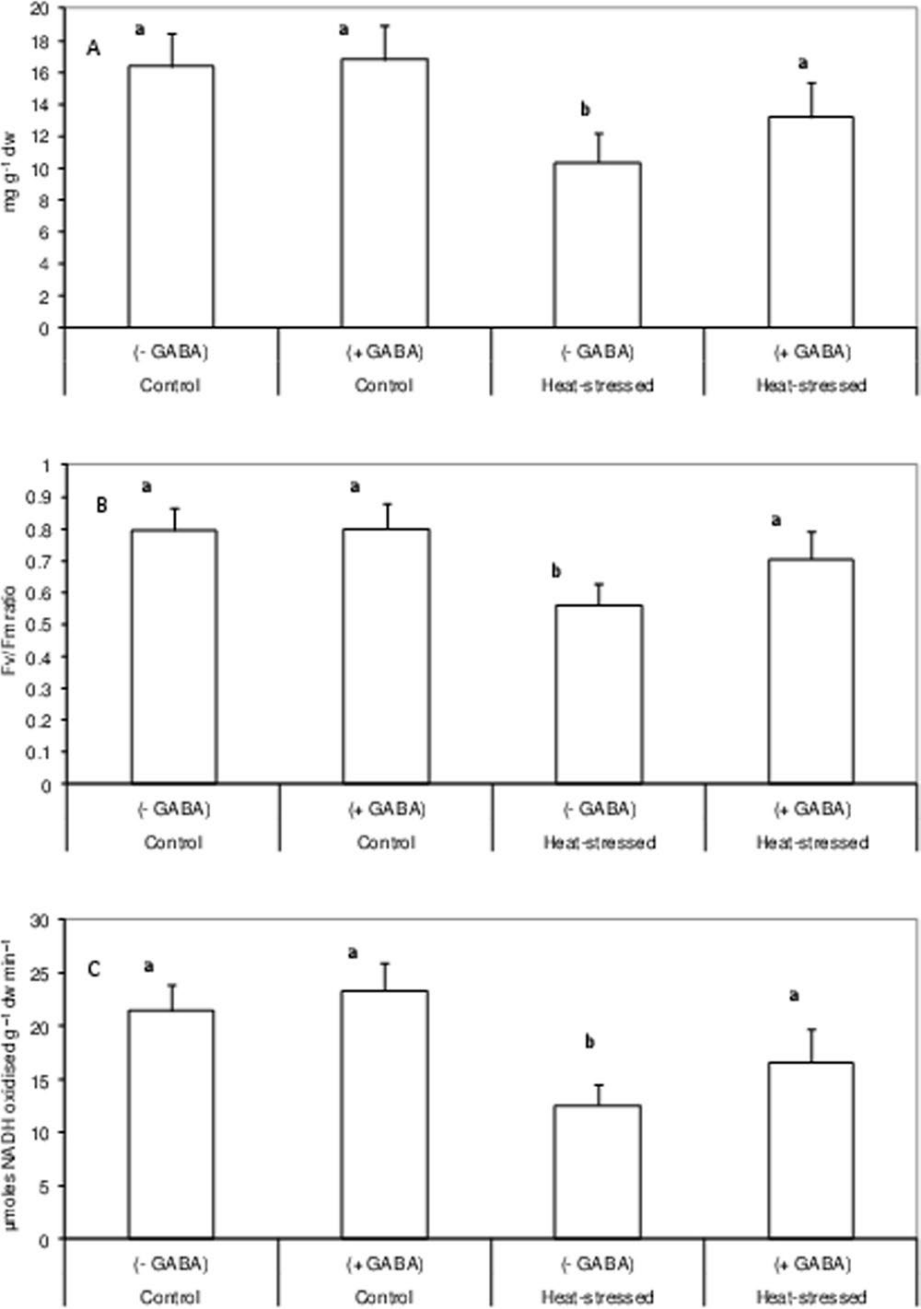
Chlorophyll concentration (**A**), PS II function (**B**) and RUBISCO activity (**C**) in leaves of control (without GABA treatment), control (with GABA treatment), heat-stressed (without GABA treatment), and heat-stressed (with GABA treatment) Mungbean plants Small vertical bars represent standard deviation (n = 3). Different alphabets on bars show significant differences (p < 0.05) from each other.

Leaf chlorophyll content declined by 37% in the heat-stress alone treatment, relative to control, but increased by 27.5% in the heat stress + GABA treatment, relative to the heat stress alone treatment (Fig. 5).

Photosystem II function, measured as leaf chlorophyll fluorescence, declined by 30% in the heat-stress alone treatment, relative to the control. The heat stress + GABA treatment increased PSII function by 26%, compared to heat-stress alone (Fig. 5).

Carbon fixation, measured as RuBisCO activity, declined by 42% in the heat-stress alone treatment, relative to the control, but recovered by 32% in the heat stress + GABA treatment (Fig. 5).

**Figure 6:**
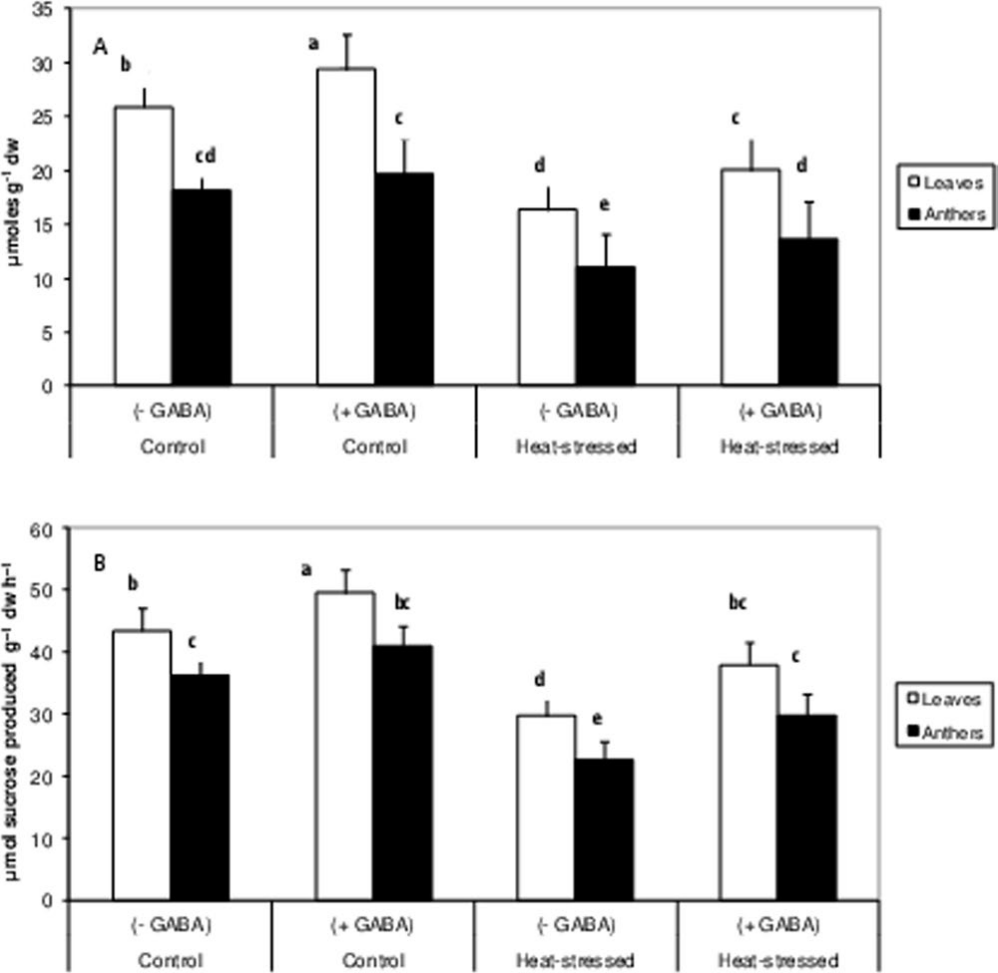
Sucrose (**A**), sucrose phosphate synthase activity (**B**) in leaves and anthers of control (without GABA treatment), control (with GABA treatment), heat-stressed (without GABA treatment), and heat-stressed (with GABA treatment) Mungbean plants. Small vertical bars represent standard deviation (n = 3). Different alphabets on bars show significant differences (p < 0.05) from each other.

Heat-stress alone decreased sucrose concentration in leaves and anthers by 36% and 39%, respectively, relative to the control alone (Fig. 6), while the heat stress + GABA treatment increased sucrose concentration by 22.5% in leaves and 24.5% in anthers, compared to heat-stress alone.

The activity of the sucrose synthesising enzyme, sucrose-P-synthase, decreased by 31.6% in leaves and 37% in anthers, in heat-stressed plants, as compared to the control (Fig. 6). The heat stress + GABA treatment increased SPS activity by 27% in leaves and 31.7% in anthers, relative to the heat-stress alone treatment. SPS activity in the control + GABA treatment was slightly but significantly higher than the control (Fig. 6).

**Figure 7:**
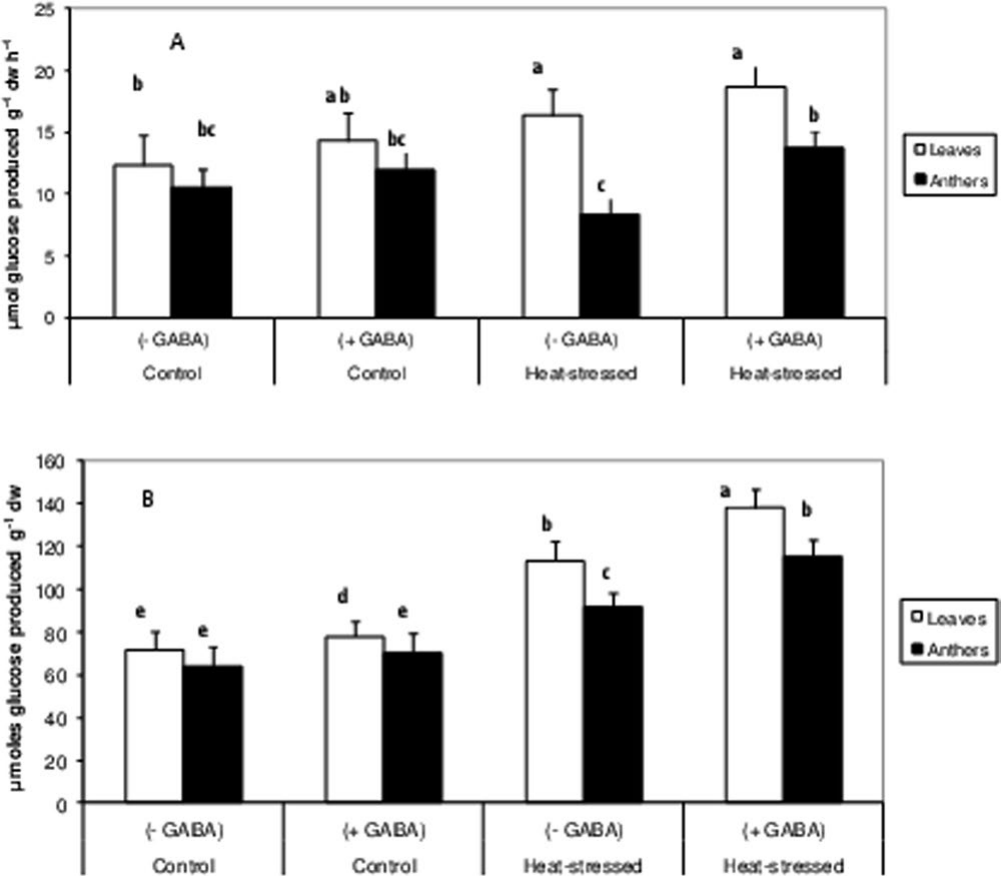
Acid invertases (vacuolar) (**A**) and reducing sugars (**B**) in leaves and anthers of control (without GABA treatment), control (with GABA treatment), heat-stressed (without GABA treatment), and heat-stressed (with GABA treatment) Mungbean plants. Small vertical bars represent standard deviation (n = 3). Different alphabets on bars show significant differences (p < 0.05) from each other.

Heat-stress alone increased acid invertase (AI) activity by 33% in leaves but decreased by 21% in anthers, relative to the control (Fig. 7). The heat stress + GABA treatment increased AI activity by 30% in leaves and 16% in anthers, relative to heat-stress alone (Fig. 6).

The concentration of reducing sugars increased by 57% in leaves and 48.5% in anthers in the heat-stress alone treatment, relative to the control (Fig. 7). The heat stress + GABA treatment increased the concentration of reducing sugars by 21% in leaves and 26% in anthers, relative to heat-stress alone.

### Oxidative stress

**Figure 8:**
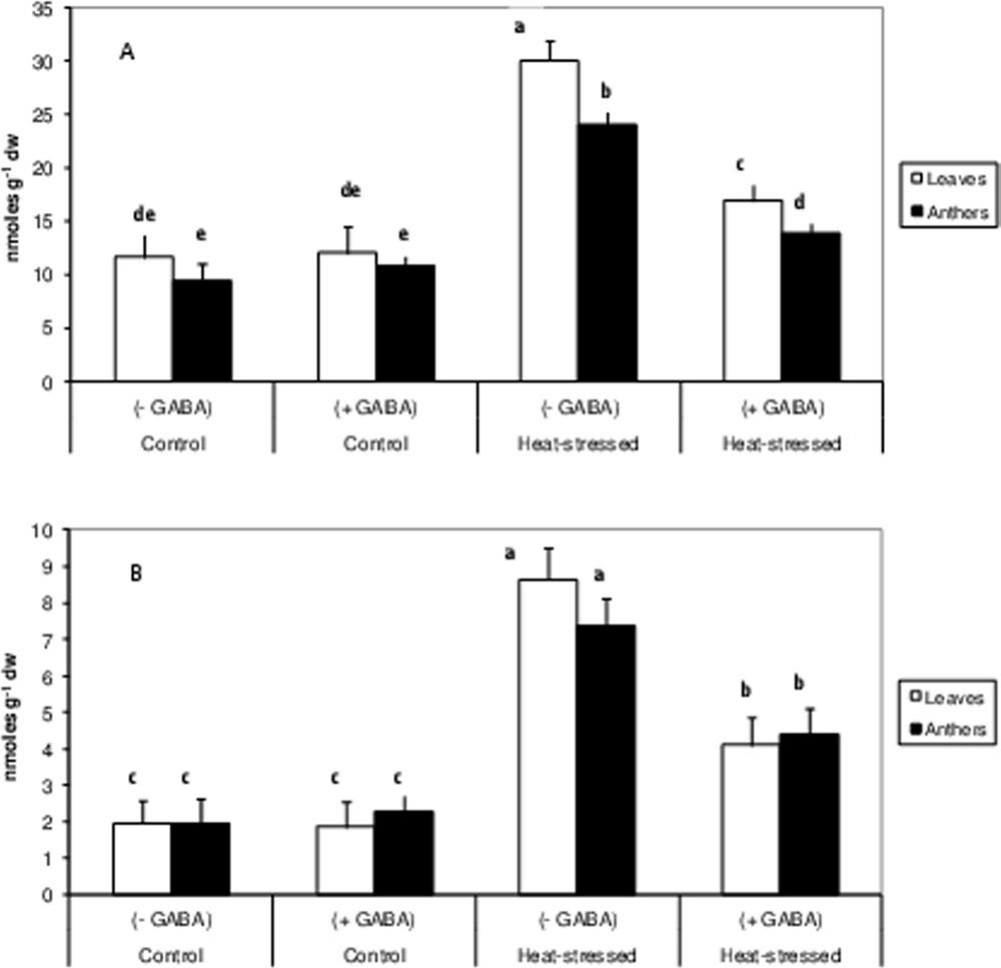
Malondialdehyde (**A**) and hydrogen peroxide concentration (**B**) in leaves and anthers of control (without GABA treatment), control (with GABA treatment), heat-stressed (without GABA treatment), and heat-stressed (with GABA treatment) Mungbean plants. Small vertical bars represent standard deviation (n = 3). Different alphabets on bars show significant differences (p < 0.05) from each other.

Oxidative damage, measured as malondialdehyde (a product of lipid peroxidation), increased about 2.6-fold in the leaves and 2.5 fold in anthers with heat-stress alone, relative to the control (Fig. 8). The heat stress + GABA treatment reduced the MDA concentration by 43.5% in leaves and 42.3% in anthers, relative to the heat-stress alone treatment.

Hydrogen peroxide (H_2_O_2_) content increased 4.4-fold in leaves and 3.7-fold in anthers with heat-stress alone, relative to the control (Fig. 8). The heat stress + GABA treatment decreased the H_2_O_2_ concentration by 52% in leaves and 40% in anthers, relative to heat-stress alone.

### Enzymatic antioxidants

**Figure 9:**
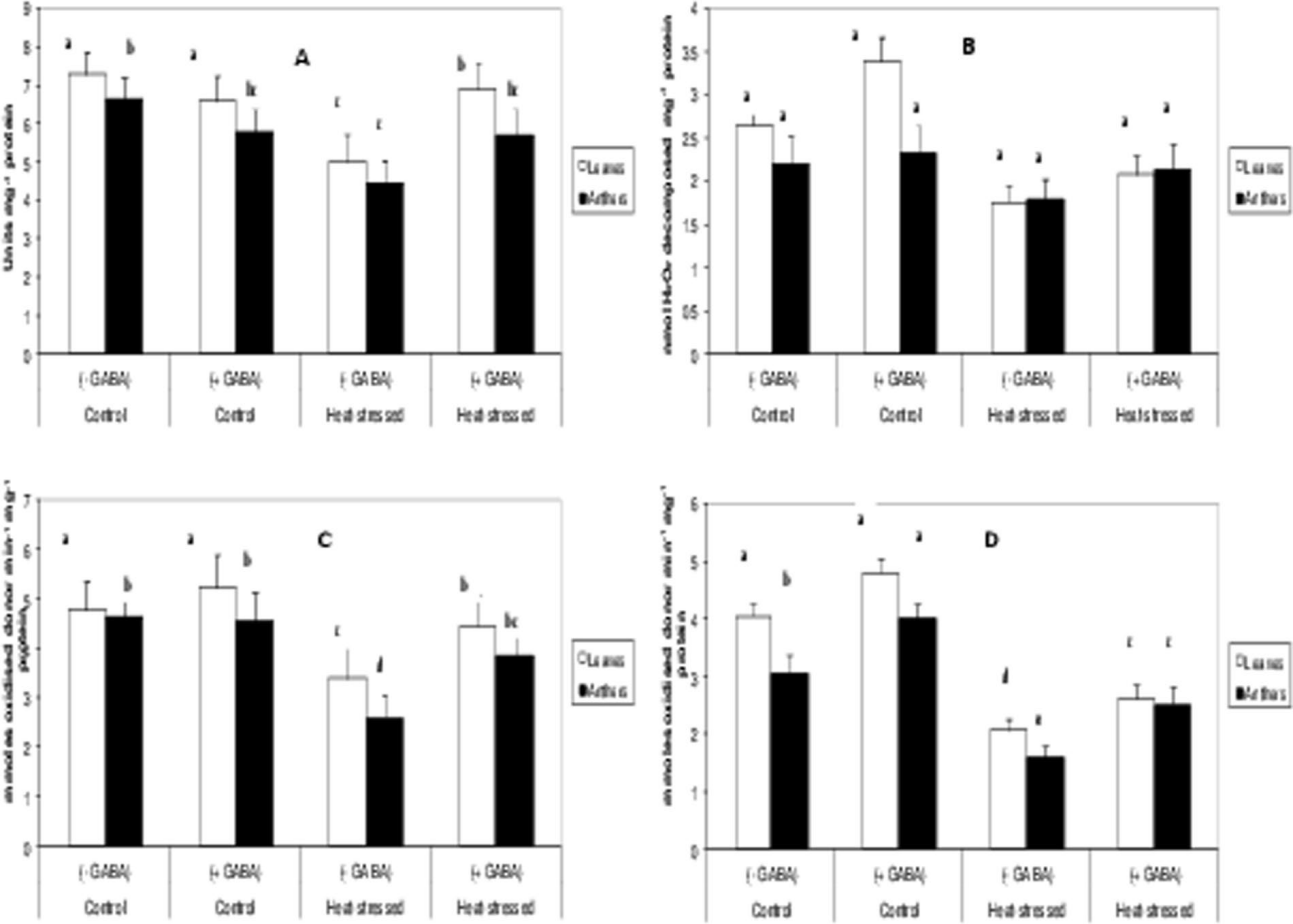
Activities of Superoxide dismutase (**A**), Catalase (**B**), Ascorbate peorxidase (**C**) and glutathione reductase (**D**) in leaves and anthers of control (without GABA treatment), control (with GABA treatment), heat-stressed (without GABA treatment), and heat-stressed (with GABA treatment) Mungbean plants. Small vertical bars represent standard deviation (n = 3). Different alphabets on bars show significant differences (p < 0.05) from each other.

Superoxide dismutase (SOD; converts superoxide to hydrogen peroxide) activity decreased by 33% in leaves and anthers with heat-stress alone, relative to the control (Fig. 9). The heat stress + GABA treatment recovered 38% of the activity in leaves and 28% in anthers, relative to the heat-stress alone treatment.

Catalase (CAT; detoxifies hydrogen peroxide) activity declined similarly to SOD in the leaves and anthers of heat-stressed plants, relative to control plants (Fig. 9). The heat stress + GABA treatment improved CAT activity by 47% in leaves and 42% in anthers, relative to heat-stress alone.

Ascorbate peroxidase (APX; detoxifies hydrogen peroxide) activity declined by 29% in leaves and 45% in anthers in the heat-stress alone treatment, relative to the control (Fig. 9). The heat stress + GABA treatment increased APX activity by 31% in leaves and 15% in anthers, relative to heat-stress alone.

Heat-stress alone reduced glutathione reductase (GR; regenerates reduced glutathione) more than the other measured enzymatic antioxidants (Fig. 9); by 48% in leaves and anthers, relative to the control. The heat stress + GABA treatment increased GR activity by 27% in leaves and 58% in anthers, relative to heat-stress alone.

### Non-enzymatic antioxidants

**Figure 10:**
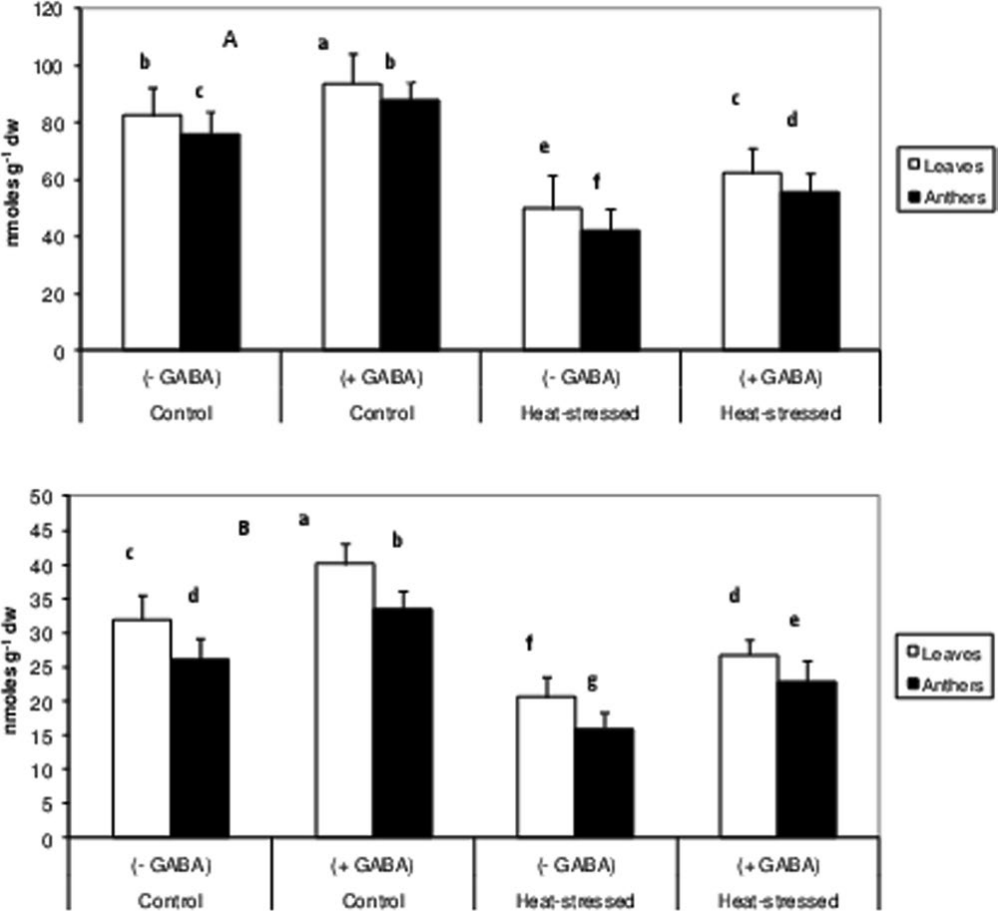
Ascorbic acid (Top) and glutathione (bottom) concentration in leaves and anthers of control (without GABA treatment), control (with GABA treatment), heat-stressed (without GABA treatment), and heat-stressed (with GABA treatment) Mungbean plants. Small vertical bars represent standard deviation (n = 3). Different alphabets on bars show significant differences (p < 0.05) from each other.

Heat-stress alone reduced ascorbate (ASC) concentration by 42% in leaves and 44% in anthers, relative to the control (Fig. 10). The heat stress + GABA treatment increased ASC concentration by 25% in leaves and 32% in anthers, relative to heat-stress alone.

Similarly, the heat-stress alone treatment decreased the GSH concentration by 35% in leaves and 38% in anthers, relative to the control (Fig. 10). The heat stress + GABA treatment increased GSH by 30% in leaves and 43% in anthers, relative to heat-stress alone.

### Osmolytes and their enzymes

#### Proline metabolism

Heat-stress alone increased the endogenous proline concentration by 1.8 fold in leaves and 1.5 fold in anthers, relative to the control (Table 1). GABA treatment to heat-stressed plants further increased the proline concentration significantly in both leaves and anthers, in comparison to heat-stress alone.

**Table 1:** Proline, trehalose concentrations and their metabolising enzymes in leaves and anthers of control (without GABA treatment), control (with GABA treatment), heat-stressed (without GABA treatment) and heat-stressed (with GABA treatment) mungbean plants.

Trait	Control (−GABA)		Control (+GABA)		Heat-stressed (−GABA)		Heat-stressed (+GABA)	
	Leaf	Anthers	Leaf	Anthers	Leaf	Anthers	Leaf	Anthers
Proline (nmoles g^−1^ DW)	16.9 ± 2.0d	15.5 ± 2.0de	20.1 ± 1.8c	17.8 ± 2.0cd	20.4 ± 2.9b	23.1 ± 2.4c	39.2 ± 2.6a	30 ± 2.8b
Proline 5 carboxylate synthase (nmol NADP formed mg^−1^ protein min^−1^	12.7 ± 2.2c	11.8 ± 2.5c	14.5 ± 2.4c	13.6 ± 2.3c	15.8 ± 2.0b	15.2 ± 1.9c	25.0 ± 2.4a	21.9 ± 2.2b
Proline dehydrogenase (nmol NADH formed mg^−1^ protein min^−1^	5.2 ± 0.78e	4.1 ± 0.84e	4.2 ± 0.58e	3.3 ± 0.24e	30.1 ± 2.5a	23.0 ± 3.0b	14.8 ± 1.8c	12.1 ± 1.6d
Trehalose (nmoles g^−1^ DW)	6.7 ± 0.78b	4.5 ± 0.66d	7.8 ± 0.84a	5.5 ± 0.73c	4.7 ± 0.73d	1.9 ± 0.23f	5.6 ± 0.35c	2.7 ± 0.47e
TPS (nmoles UDP mg^−1^ protein min^−1^	48.4 ± 3.4b	35.4 ± 3.0d	55.8 ± 3.9a	39 ± 4.1c	27.1 ± 3.0f	22.5 ± 1.8g	41.4 ± 3.5c	32.0 ± 3.7e
TPP(nmoles UDP min^−1^ mg^−1^ protein	26.1 ± 2.7b	20.9 ± 2.1d	31.6 ± 2.7a	22.4 ± 2.0d	20.1 ± 1.8d	14.3 ± 1.4f	23.0 ± 2.5c	17.3 ± 2.0e
Trehalase (µmol glucose min^−1^ mg protein^−1^	24.3 ± 2.6a	21.8 ± 2.3b	29.8 ± 2.7a	23.5 ± 2.8b	24.5 ± 3.0b	19.9 ± 2.4c	20.9 ± 2.7c	16.9 ± 2.4d

Mean ± SD (n = 3). Different letters along with values in a row indicate significant differences (p < 0.05).

The activity of proline-5-carboxylate synthase (P5CS), which synthesises proline, increased by 24% in leaves and 34% in anthers in the heat-stress alone treatment, relative to the control (Table 1). The heat stress + GABA treatment increased P5CS activity by 58% in leaves and 38% in anthers, relative to heat-stress alone, without GABA.

The enzyme activity of proline dehydrogenase (PDH; catabolises proline; PDH) increased by 80% in leaves and 14.4% in anthers in heat-stress treatment alone, compared to control. The heat stress + GABA treatment decreased PDH activity by 28% in leaves and 29.5% in anthers, relative to heat-stress alone (Table 1).

#### Trehalose metabolism

Trehalose concentration, in the heat-stress alone treatment, decreased by 30% in leaves and 56% in anthers, relative to the control (Table 1). The heat stress + GABA treatment increased trehalose levels by 19% in leaves and 38% in anthers, relative to heat-stress alone.

The activity of trehalose-6-phosphate synthase (TPS), the first enzyme in trehalose biosynthesis, which generates precursor (trehalose-6-phosphate) of trehalose, using glucose-6-phosphate and UDP-glucose, declined by 44% in leaves and 37% in anthers in the heat-stress alone treatment, relative to the control (Table 1). The heat stress + GABA treatment resulted in a remarkable recovery of TPS activity (52% in leaves and 45% in anthers), relative to heat-stress alone. The control + GABA treatment also resulted in a small but significant increase in TPS activity, relative to the control.

The activity of trehalose-6-phosphate phosphatase (TPP), the second enzyme in trehalose biosynthesis, which dephosphorylates TPP to produce trehalose, declined by 23% in leaves and 32% in anthers, relative to the control (Table 1). The heat stress + GABA treatment increased TPP activity by 17% in leaves and 20% in anthers, relative to heat-stress alone.

The activity of trehalase (hydrolyses trehalose) decreased slightly in leaves and anthers in the heat-stress alone treatment, relative to the control (Table 1). The heat stress + GABA treatment reduced trehalase activity (by 15% in leaves and anthers), relative to the heat-stress alone.

#### Yield traits

The control plants had 76% pod set, which increased to 85.3% with GABA application. Heat-stress alone reduced pod set to 36%, while the heat stress + GABA treatment increased pod set to 59% (Table 2).

**Table 2:** Yield traits of control (without GABA treatment), control (with GABA treatment), heat-stressed (without GABA treatment) and heat-stressed (with GABA treatment) mungbean plants.

Trait	Control (−GABA)	Control (+GABA)	Heat-stressed (−GABA)	Heat-stressed (+GABA)
Pod set (%)	76.4 ± 5.7b	85.3 ± 4.7a	36.2 ± 5.3d	59.5 ± 5.8c
Pod number plant^−1^	12.2 ± 1.9b	14.5 ± 1.7a	5.6 ± 1.5d	7.1 ± 1.5c
Average pod length (cm)	9.4 ± 1.3a	9.0 ± 1.5a	5.2 ± 1.6c	6.6 ± 1.6b
Pod yield plant^−1^	6.9 ± 1.7a	7.3 ± 1.6a	3.3 ± 0.82b	4.5 ± 0.73b
Seed yield plant^−1^	5.0 ± 0.91a	5.2 ± 0.80a	2.6 ± 0.48c	3.3 ± 0.54b

Mean ± SD (n = 3). Different letters along with values in a row indicate significant differences (p < 0.05).

Heat-stress alone reduced pod number (plant^−1^) by 54%, pod yield (g plant^−1^) by 52%, pod length per plant by 46% and seed yield by 48%, relative to the control (Table 2). The heat stress + GABA treatment improved pod number by 28%, pod yield by 37%, pod length by 25.7% and seed yield by 27%, relative to heat stress alone.

## Discussion

Heat stress caused a substantial loss of flowers and pods in mungbean plants, which confirmed our findings in the previous study^6^. Reproductive function, assessed as pollen and stigmatic function, declined markedly in heat-stressed mungbean plants, which is similar to previous observations in mungbean^6^, chickpea^18^ and lentil^7^. The present study assessed the effectiveness of GABA in protecting the reproductive function of mungbean under heat stress. In preliminary studies, we noticed that the endogenous levels of GABA in leaves and anthers declined considerably as the heat stress progressed (unpublished data). This was associated with substantial flower loss and the failure of many flowers to become pods, suggesting impaired fertilisation. Hence, we hypothesised that depletion of endogenous GABA levels in vegetative (leaves) as well as reproductive (anthers) components under heat stress might be a critical factor affecting reproductive function. GABA has been implicated as an important amino acid in sexual reproduction in angiosperms^16^, and maintenance of its adequate levels is vital for post-pollination development^16^. GABA has also been implicated in response to various abiotic stresses^11^, including heat stress^10^. To substantiate our hypothesis, we exogenously supplemented GABA (1 mM) through seed priming and foliar sprays, which markedly increased the endogenous GABA in leaves and anthers and significantly improved pollen germination, pollen viability, stigma receptivity and ovule viability, which reduced flower abortion and improved pod set, pod number and seed yield in heat-stressed mungbean plants. Previously, GABA has enhanced plant growth at concentrations of 250 μM in *Stellaria longipes*^19^ and 5 μM in *Lemna*^13^ under no stress and 1 mM in laboratory-grown rice plants^10^ and 0.5 mM in creeping bentgrass (*Agrostis stolonifera*)^12^ under heat stress. Our study has, for the first time, demonstrated the involvement of GABA in protecting reproductive function from heat stress in mungbean plants.

We examined the mechanisms through which GABA might protect reproductive components of mungbean against heat stress. We investigated vegetative structures (leaves) and reproductive components (anthers) in this regard. Since the leaves provide all the plant’s nutritional requirements, any leaf damage would limit the development and function of reproductive components. The leaves of heat-stressed mungbean plants lost membrane integrity, as observed in other heat-stressed plant species such as maize, rice^20^, chickpea^18^ and lentil^21^, and may be due to direct^22^ or indirect^23^ effects involving oxidation. GABA-treated mungbean plants had less membrane damage, which could be related to the enhanced the leaf water status and reduced oxidative stress, and is in accordance with earlier studies in heat-stressed rice^10^ and creeping bentgrass^12^.

Leaf water content decreased significantly in heat-stressed plants, which could be linked to decrease in stomatal conductance and/or hydraulic root conductivity^24^. Moreover, heat stress inhibited the production of osmolytes, such as proline and trehalose, in mungbean, which might have decreased osmoregulation ability and leaf turgor^25^. GABA-treated mungbean plants accumulated proline and trehalose, which was related to the up-regulation of biosynthetic enzymes and down-regulation of catabolising enzymes, and agrees with our previous study in heat-stressed rice^10^. The present study identified stimulatory effects of GABA on enzymes related to proline and trehalose biosynthesis; this is the first such report in heat-stressed plants. In a salt-stressed tomato cultivar, GABA accumulation preceded the accumulation of proline and sugars, suggesting its role in signalling in affecting the biosynthesis of these osmolytes^26^. Moreover, GABA has been suggested to act as an osmolyte^27^; an increase in the endogenous GABA concentration in GABA-treated plants might improve the osmoregulation ability of heat-stressed plants. Cellular viability decreased in heat-stressed mungbean plants indicating a loss of mitochondrial function, which is likely due to the denaturation or inactivation of enzymes involved in respiratory metabolism. The reduction in cell viability matches observations in maize, rice^20^, lentil^21^ and chickpea^18^ subjected to heat stress. GABA application restored cellular viability in heat-stressed mungbean plants suggesting the stability of mitochondrial function in leaves and anthers, which might be related to improved leaf water status.

Oxidative stress is one of the vital negative effects of heat stress, which is reflected in malondialdehyde (MDA) and hydrogen peroxide (H_2_O_2_) concentrations. Heat stress increased MDA and H_2_O_2_ concentrations several-fold in the leaves and anthers of mungbean, which would intensify membrane and cellular damage^28^, and agrees with other studies on heat-stressed chickpea^29^ and lentil^7^. Oxidative stress is a as a result of damage to chloroplasts and mitochondria by high temperatures^30^. Symptoms such as chlorosis, necrosis and bleaching due to heat stress, as observed in our study, could be related to oxidative damage in leaf tissues^31^. Cells regulate their redox status by activating various enzymatic and non-enzymatic antioxidants^28^. We observed reduced expression of these antioxidants in heat-stressed mungbean plants, which likely aggravated the oxidative damage. Our findings are in accordance with observations in heat-stressed chickpea^31^, rice and maize^20^, where cellular damage was related to a marked reduction in various antioxidants. In contrast, GABA-treated heat-stressed plants showed significantly less oxidative damage, as revealed by marked reductions in MDA and H_2_O_2_ concentrations in leaves and anthers. These reductions were connected with enhanced activity levels of various antioxidants in leaves and anthers, which significantly reduced membrane damage in these organs and chlorophyll in leaves. Previous studies have shown an increase in anti-oxidative capacity in GABA-treated plants in response to heat stress in rice^10^, salt stress^11^, and cold stress in peach plants^32^. GABA appears to up-regulate the antioxidant system by some unknown mechanism, which needs to be investigated. Previous studies have revealed GABA as a signalling molecule that activates enzymes such as arginine decarboxylase, as in soybean^33^, and induce expression of genes related to nitrate uptake in *Brassica napus*^34^.

Leaf chlorosis in heat-stressed mungbean plants occurred due to a substantial reduction in chlorophyll, which also adversely impacted PSII function and RuBisCO activity to impair leaf photosynthesis. The reduction in chlorophyll with high temperature might have resulted from inhibited chlorophyll biosynthesis or increased chlorophyll degradation^35^, and/or disorganisation of chloroplasts due to photooxidation^36^. Other heat-stress studies have reported chlorophyll damage in tomato^37^, chickpea^38^ and mungbean^6^. In our study, RuBisCO activity might have been inhibited due to a reduction in stomatal conductance, or other reasons such as decreased rates of RuBP regeneration, as a result of the impaired electron transport activity, especailly, the inactivation of the oxygen-evolving enzymes of PSII^39^. Moreover, carbon assimilation decreased in heat-stressed mungbean plants, as indicated by reductions in SPS activity and sucrose concentration in leaves and anthers, which might explain the negative effects of heat stress on reproductive components and their function^40^. At the same time, acid invertases (vacuolar) activity declined significantly in leaves and anthers of heat-stressed plants. Acid invertases hydrolyse sucrose to simple hexoses, which are used by cells for multiple purposes, and work in tandem with SPS to maintain the supply of hexoses under normal situations. In this study, a reduction in acid invertases indicated the disruption of sucrose metabolism, and therefore reproductive function^40,41^. The heat stress + GABA treatment significantly increased the sucrose concentration in leaves and anthers of mungbean and was attributed to up-regulation of the activities of RuBisCO and SPS. At the same time, the activity of acid invertases increased, which possibly facilitated the availability of hexoses to anthers and other reproductive components to restore flower function and contribute to improved pod set and other yield-related traits. How GABA regulates the activities of sucrose synthesising enzymes needs to be probed further.

In conclusion, this study identified that heat-stressed mungbean plants treated with GABA increased carbon fixation and assimilation to enhance sucrose synthesis in leaves, and possibly its transport to flowers, to sustain reproductive function, which increased the retention of flowers and pods, thus suggesting the effectiveness of GABA as a thermo-protectant. Moreover, GABA application to heat-stressed plants improved leaf water status, probably by up-regulating the enzymes related to the synthesis of osmolytes such as proline and trehalose. At the same time, it reduced the oxidative damage to the reproductive components. GABA supplementation may be beneficial for developmental and functional aspects of flowers, related to reproductive function, and needs further investigation in stressed plants. To our knowledge, this is the first report identifying the importance of GABA in protecting the reproductive function of mungbean plants against high-temperature stress. This information paves the way for generating new insights into functional aspects of the GABA.

## Methods

### Raising of plants

The seeds of mungbean genotype (SML 668 sourced from Punjab Agricultural University, Ludhiana, India) were primed for 6 h with 1 mM GABA and planted in earthenware pots having a combination of air-dried soil, sand and farmyard manure, in proportions of 2:1:1 (v/v), at Panjab University, Chandigarh (30.7333°N, 76.7794°E), India. The soil had a pH of 7.1, loamy in texture, having available N, P, and K, @ 54,43 and 158 kg ha^−1^, respectively. The primed seeds were inoculated with *Rhizobium* sp. (@1.95 g kg^−1^). In each pot, 3 seeds were planted in March (last week), which, after emergence, were subsequently thinned to 2 per pot. These plants were raised in in a wired enclosure, under natural, outdoor environmental condtions (29.3/16.1 ± 1 °C mean day/night temperature, 1350–1550 µmol m^−2^ s^−1^ light intensity, 60–65% mean relative humidity; Fig. 1) until the commencement of the reproductive stage (30 days after sowing). Thereafter, half of the plants were maintained in a controlled environment at 35/23 °C (control) while the other half were subjected to heat stress. For the heat-stress treatment, the plants were initially exposed in a controlled environment for one day each to 38/28, 40/30, 42/30 °C, and then maintained at 45/30 °C; these temperatures are likely the high temperatures faced in the field. The heat-stress at 45/30 °C was provided until maturity. All plants, including controls, received about 800 µmol m^−2^ s^−1^ light intensity and 65–70% relative humidity to maturity. The plants were kept fully irrigated during the course of study to avoid any drought stress.

### Standardisation of GABA treatments and concentrations

In preliminary experiments, GABA was applied to 30-day old mungbean plants, at various concentrations, as 0.5, 0.75, 1.0, 1.5, and 2 mM, through root drenching once (during irrigation), 2 days before exposure to heat stress, and foliar sprayed (along with Tween 20, as a sticking agent) at the same time. Another GABA foliar treatment was provided four days after heat stress (exposure to 45/30 °C). Application of 1 mM GABA (both as a root drenching treatment and foliar spray) exerted maximum benefits for yield traits (pod number, seed number and seed weight) in heat-stressed mungbean plants (data not shown).

Hence 1 mM GABA was used for subsequent experiments, which was applied in a similar way, as above (one root drenching and 2 foliar treatments). Finally, there were following treatments for separate experiments: (a) control (no heat stress or GABA), (b) control + 1 mM GABA, (c) heat-stress alone, and (d) heat stress + 1 mM GABA. The following observations were recorded.

### Endogenous GABA

GABA concentration during the stress period was estimated following the method of Saito *et al*.^42^. After 15 days, leaves and anthers were collected from the control and stressed plants (as per the treatments above), and snap-frozen. Subsequently, the tissue (500 mg) was extracted in 8% (m/v) trichloroacetic acid (TCA), followed by centrifugation at 10,000 rpm for 20 min at 25 °C. The supernatant was collected in a new tube, and pure diethyl ether (400 ml) was included, followed by thorough mixing for 10 min. The mixture was centrifuged again for 20 min at 10,000 rpm. The supernatant was collected, and 400 ml of diethyl ether was added, mixed thoroughly for 10 min, and subsequently centrifuged for 10 min at 10,000 rpm. The supernatant was allowed to stand for 30 min under a draft of air, for complete evaporation of ether. The ‘GABase’ assay evaluates the reduction of NADP to NADPH, using GABA (Sigma) as a substrate (pH 8.6, at 25 °C), as a function of time, spectroscopically at 340 nm.

### Analysis of stress injury to leaves

The fresh leaves (from the second and third top branches) and flowers (for anthers) were collected from the control and heat-stressed plants (at 11:00 h) after 15 days of treatment and analysed for various parameters. Flowers and leaves were harvested from five plants per treatment in three replications (15 plants per treatment) for analysis.

The fresh leaf tissue was assessed for membrane damage on the basis of electrolyte leakage (EL). The leaf tissue was analysed for injury to membranes using electrolyte leakage as indicator^43^. The leaf water status of the top most leaves was measured following the method of Barrs and Weatherley^44^.

Chlorophyll was analysed from fresh leaves, harvested from control and heat-stressed plants, according to the method of Arnon *et al*.^45^.

PS-II activity was analysed at 11:00 h^40^ from the same leaves, using chlorophyll fluorescence, on the basis of dark-adapted test, by means of a modulated chlorophyll fluorometer (OS1-FL, Opti-Sciences, Tyngsboro, MA, USA).

The stomatal conductance (g_s_) from fully expanded leaves (from the second or third branches from the top), was measured at the same time, by a portable leaf porometer (model SC1, Decagon Devices, Pullman, WA, USA), and expressed as mmol m^−2^ s^−1^ ^40^. For measring these traits, 4 plants in 3 replications (Total 12 plants per treatment) were used. The same plants were used for assessing the seed yield.

### Carbon fixation and assimilation enzymes, sucrose and reducing sugars

The tissue samples collected were snap-frozen for assaying the activity of various enzymes, subsequently. The extraction assay for RuBisCO activity used the method of Wang *et al*.^46^, with the activity estimated as per Racker^47^. For assays of sucrose synthase and acid invertase, fresh samples (~500 mg, 3 replications) were snap-frozen, and later extracted in ice-cold 200 mM HEPES/KOH buffer (pH 7.8) having 1% (w/v) polyvinylpyrrolidone (PVP), 10 mM dithiothreitol (DTT), 3 mM magnesium acetate, 3 mM EDTA Na_2_.2H_2_O. This was followed by centrifugation of the homogenate for 20 min at 4 °C at 10,000 rpm; the supernatant acted an enzyme and protein source. Subsequently, desalting of the supernatant was done by passing it through 4 mL Sephadex G-25 columns (Sigma, St Louis, MO, USA). These columns have been pre-equilibrated with a buffer solution having 20 mM HEPES–NaOH (pH 7.5), 0.05% BSA, 1 mM EDTA, 0.01% 2-mercaptoethanol, 0.25 mM MgCl_2._, followed by assaying of the desalted extract^47^. The activity of sucrose synthase activity was assayed according to Hawker *et al*.^48^, while that of Vacuolar acid invertase was measured, according to the method of Nygaard^49^. The concentration of sucrose was analysed, as per the enzymatic method of Jones *et al*.^50^, while the concentration of reducing sugars was assessed using DNSA method^51^. The biochemical analysis was performed in 3 plants in 3 replications (Totally nine plants per treatment).

### Reproductive function

Pollen grains, gathered from the control and heat-stressed plants, were tested for viability (in 5–10 microscopic fields) using 0.5% acetocarmine/Alexander stain^18^. The pollen load and pollen germination (*in vivo*) were assessed in the flowers (gathered, as above, for testing pollen viability) having fully-dehiscent anthers. Pollen load (refers to intensity of pollen on stigma surface) was evaluated on the basis of 1–5 scale^52^ (1 = low and 5 = high). At the same time, germinated and non-germinated pollen grains (number) on the stigma surface were examined in control and heat-stressed flowers^18^. Pollen grains were tested for *in vitro* germination; pollen were harvested from five flowers per genotype in three replications. The germination was assessed, according to the method of Brewbaker and Kwack^53^ with the help of a medium having 10% sucrose, potassium nitrate (990 mM), magnesium sulphate (812 mM), calcium nitrate (1,269 mM), boric acid (1,640 mM; pH 6.5). Pollen grains were considered as germinated, when the tube size surpassed the diameter of the pollen grain. At least 100 pollen grains per replicate were tested for this purpose^18^.

Stigma receptivity was detected using an esterase test, which involved α-naphthyl acetate as a substrate linking the azo-coupling reaction with fast blue B, as per the modifications in the method of Mattson *et al*.^54^. Ovule viability was evaluated using 2,3,5-triphenyl-2 H-tetrazolium chloride (TTC) reduction test^18^.

### Effect of GABA on pollen germination (*in vitro*)

Pollen grains, collected from control plants, were germinated (as per the method described above in reproductive function) in a growth medium supplemented with GABA (1 mM) at varying temperatures (in controlled environment).

### Oxidative molecules and antioxidants

The plant tissue was collected and snap-frozen for analysis of concentration of various molecules and activities of enzymes. Malondialdehyde (MDA) concentration was measured to assess lipid peroxidation of membranes, as per the method of Heath and Packer^55^. Another molecule related to oxidative stress, hydrogen peroxide, was measured using the method of Mukherjee and Chaudhari^56^.

Activity of superoxide dismutase (E.C. 1.15.1.1) was assayed according to the method of Dhindsa *et al*.^57^, while for assaying catalase activity, the method of Teranishi *et al*.^58^ was used with some modifications. Activity of ascrobate peroxidase (APX; E.C. 1.11.1.11) activity was assayed by following the oxidation of ascorbate, as a reduction in absorbance at 290 nm^59^. Glutahione reductase (E.C. 1.6.4.2) activity was assayed as per the procedure of Mavis and Stellwagen^60^.

Estimation of ascorbic acid (ASC) was performed following the method of Mukherjee and Chaudhari^56^, while reduced glutathione (GSH) was estimated according to the method of Griffith^61^.

### Osmolytes and related enzymes

The extraction of proline was done from the tissues by means of 3% sulphosalicylic acid, and measured by developing a reaction with acidic ninhydrin reagent^62^.

To assess proline metabolising enzymes, tissue samples were harvested and snap-frozen, followed by homogenization in 0.1 M potassium phosphate buffer (pH 7.5) containing 1% (m/v) polyvinylpyrrolidone, 0.6 M KCl, 5 mM MgCl_2_, 10 mM mercaptoethanol, 1 mM EDTA and in a pre-chilled pestle and mortar. The homogenate was centrifuged at 4 °C for 30 min at 10,000 rpm. The supernatant was used to assess proline metabolising enzymes. Pyrroline-5-carboxylate synthase (P5CS) activity was assayed by the method described by Filippou *et al*.^63^. Proline dehydrogenase (PDH) was assayed following the NADP reduction at 340 nm in 0.15 M Na_2_ CO_3_ buffer (pH 10.3) having 1.5 mM NADP, 15 mM proline^64^.

Trehalose was estimated as per method of Trevelyan and Harrison^65^ and Anthrone method of Brin^66^. The enzymes related to trehalose metabolism were assayed as per the methods given in Pramanik & Imai^67^ with slight modifications.

Activity of trehalose-6-phosphate synthase (TPS) was assayed following the method of Hottiger *et al*.^68^, which in the presence of glucose-6-phosphate, estimates the release of UDP from UDP-glucose.

The activity of Trehalose-6-phosphate phosphatase (TPP) was assayed following the method of Klutts *et al*.^69^, which measured the the release of inorganic phosphate from trehalose-6-phosphate.

Neutral trehalase activity was assayed involving its activation by phosphorylation via cAMP (cyclic adenosine monophosphate), and assayed by determination of glucose^70^.

### Yield traits

Ten plants in 3 replications (Total 30 plants per treatment) were examined. Pod numbers, seed number, seed weight (per plant basis) were recorded at maturity in control and stressed plants. No destructive assay was performed on these plants.

The experiment was conducted partly outdoors (till flower initiation) and in a controlled environment (for exposure to heat stress) in a growth chamber.

The experiment was repeated twice over two years, the average values of the data of 2 years are presented.

### Statistical analysis

The experiments were conducted using a randomised block design. For each trait, observations were replicated thrice, and data were computed for calculating means and standard errors. ANOVA was pefromed, and least significant values (LSD) values were determined (p < 0.05). To compare the differences between the mean values, Tukey’s post-hoc test was applied.
